# Supplementary figures and images for: Complementary antibody lineages achieve neutralization breadth in an HIV-1 infected elite neutralizer
Source: PLoS Pathog. 2022 Nov 17;18(11):e1010945. doi: 10.1371/journal.ppat.1010945 (PMC9714913; doi:10.1371/journal.ppat.1010945)

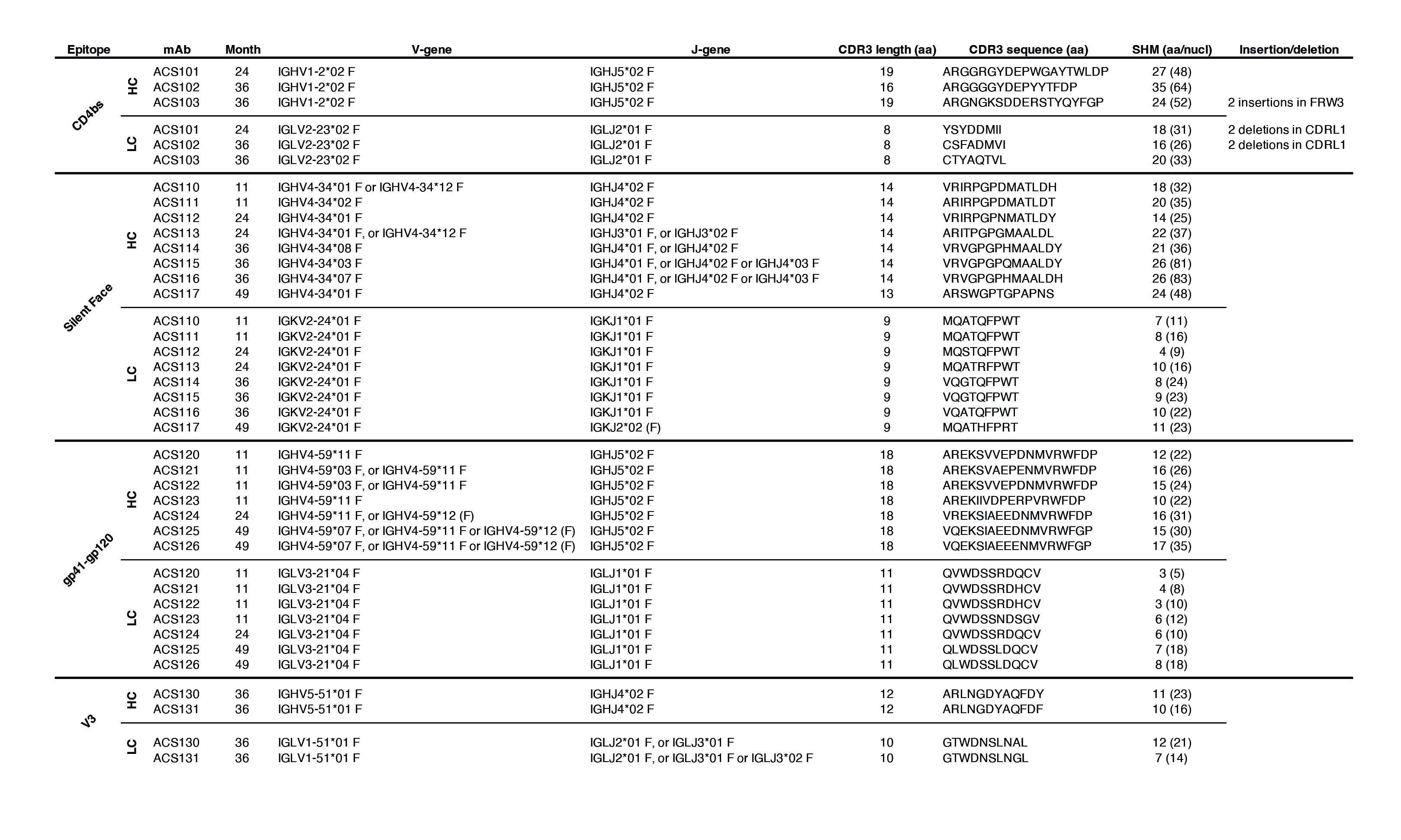

Supplement: S1 Fig — Somatic hypermutation (SHM) is determined as the percentage of nucleotide differences with the VH, VL and VK gene segments. (TIF) [file ppat.1010945.s001.tif]

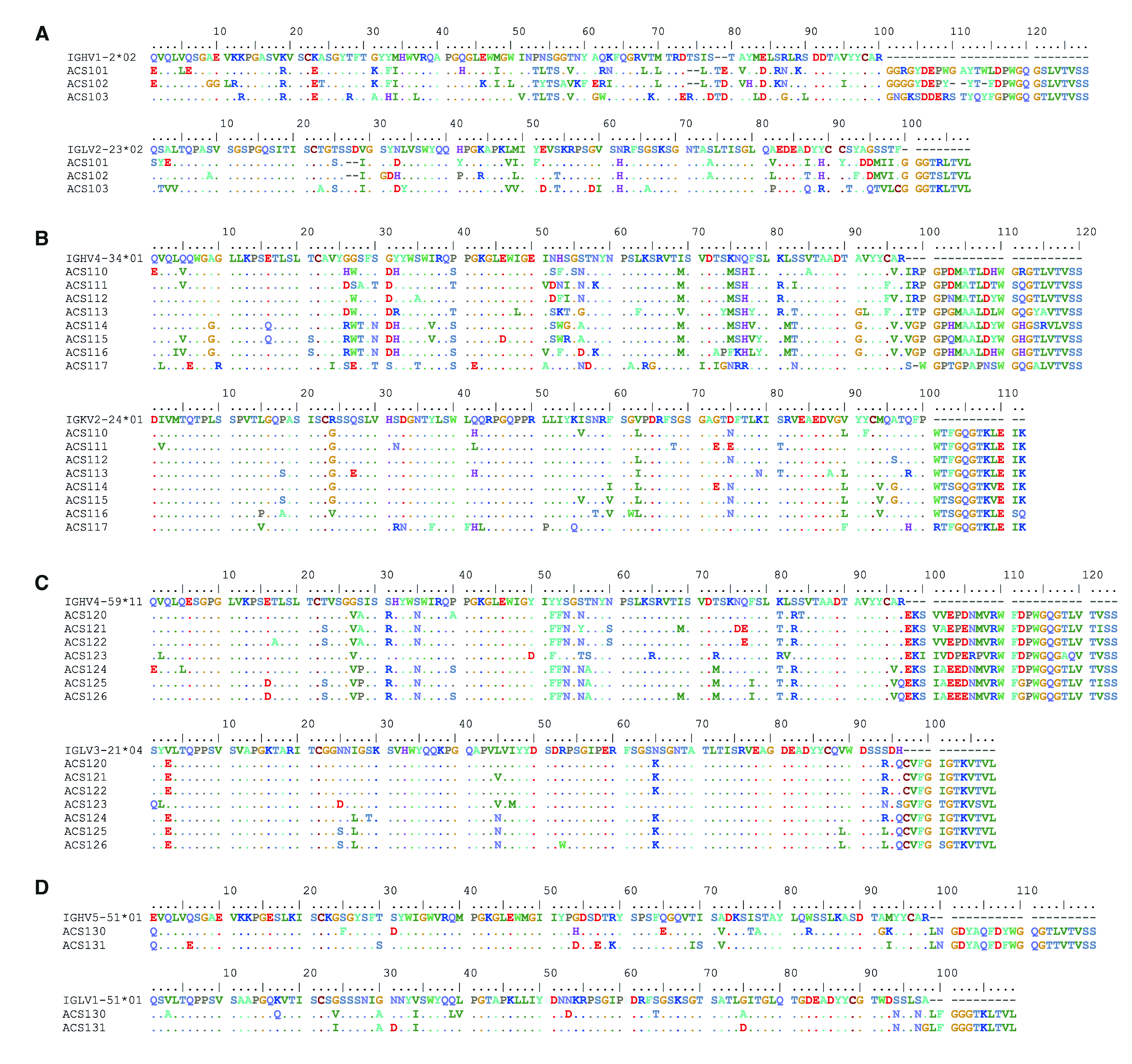

Supplement: S2 Fig — Antibody heavy and light chain sequence comparisons to the VH, VL or VK germline gene segments for mAbs targeting the (a) CD4-binding site, (b) silent face, (c) gp120-gp41 interface and (d) V1/V1/V3 region. (TIF) [file ppat.1010945.s002.tif]

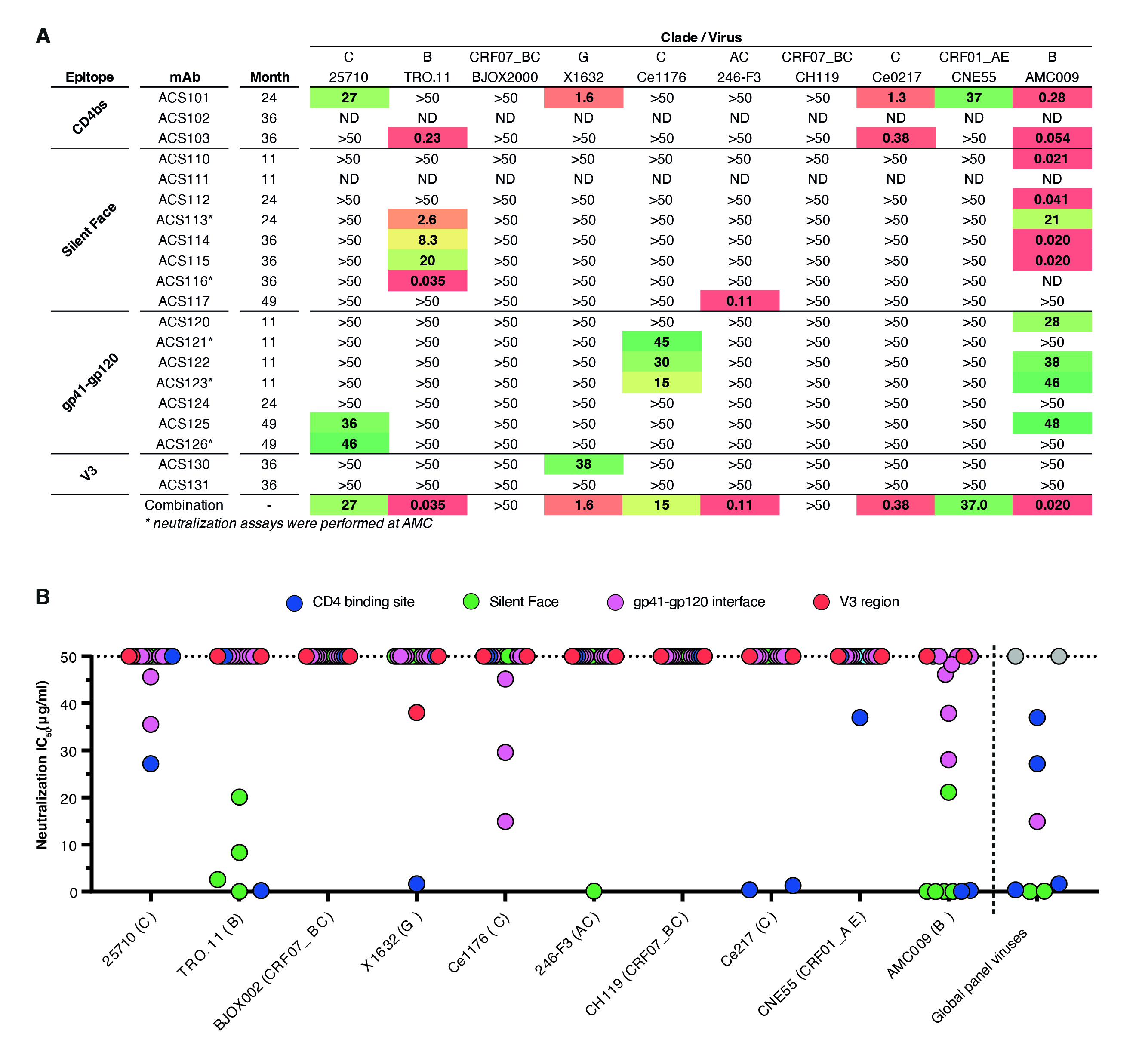

Supplement: S3 Fig — (a) Neutralization potency (antibody concentration (μg/ml) that inhibits 50% of viral infectivity (IC50) (μg/ml) is presented in table format. The mAbs were tested at a starting concentration of 50 μg/ml. (b) Neutralization potency (antibody concentration (μg/ml) that inhibits 50% of viral infectivity (IC50)) is depicted as a dot plot. The tested viruses are indicated along the horizontal axis. Each symbol represents a mAb with the different colors indicating the epitope targeted. We also provide the estimated neutralization breadth if the most potent NAbs were combined and tested against the global panel viruses. (TIF) [file ppat.1010945.s003.tif]

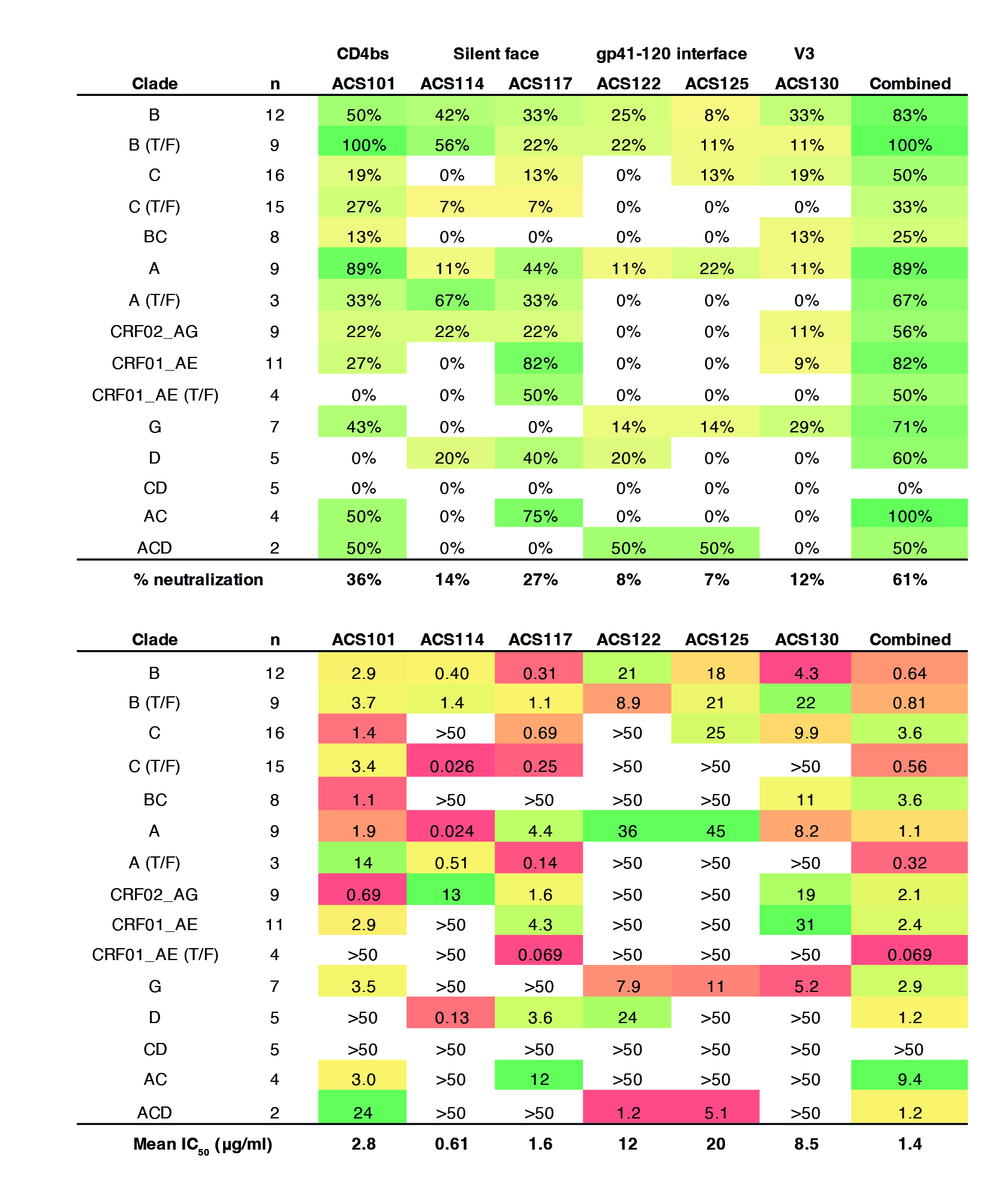

Supplement: S4 Fig — Breadth is indicated below as the percentage of viruses that were neutralized (upper panel). Geometric mean of the IC50 (μg/ml) is given as the antibody concentration that inhibits 50% of viral infectivity (lower panel). We also provide an estimated neutralization breadth/IC50 of all mAbs combined. (TIF) [file ppat.1010945.s004.tif]

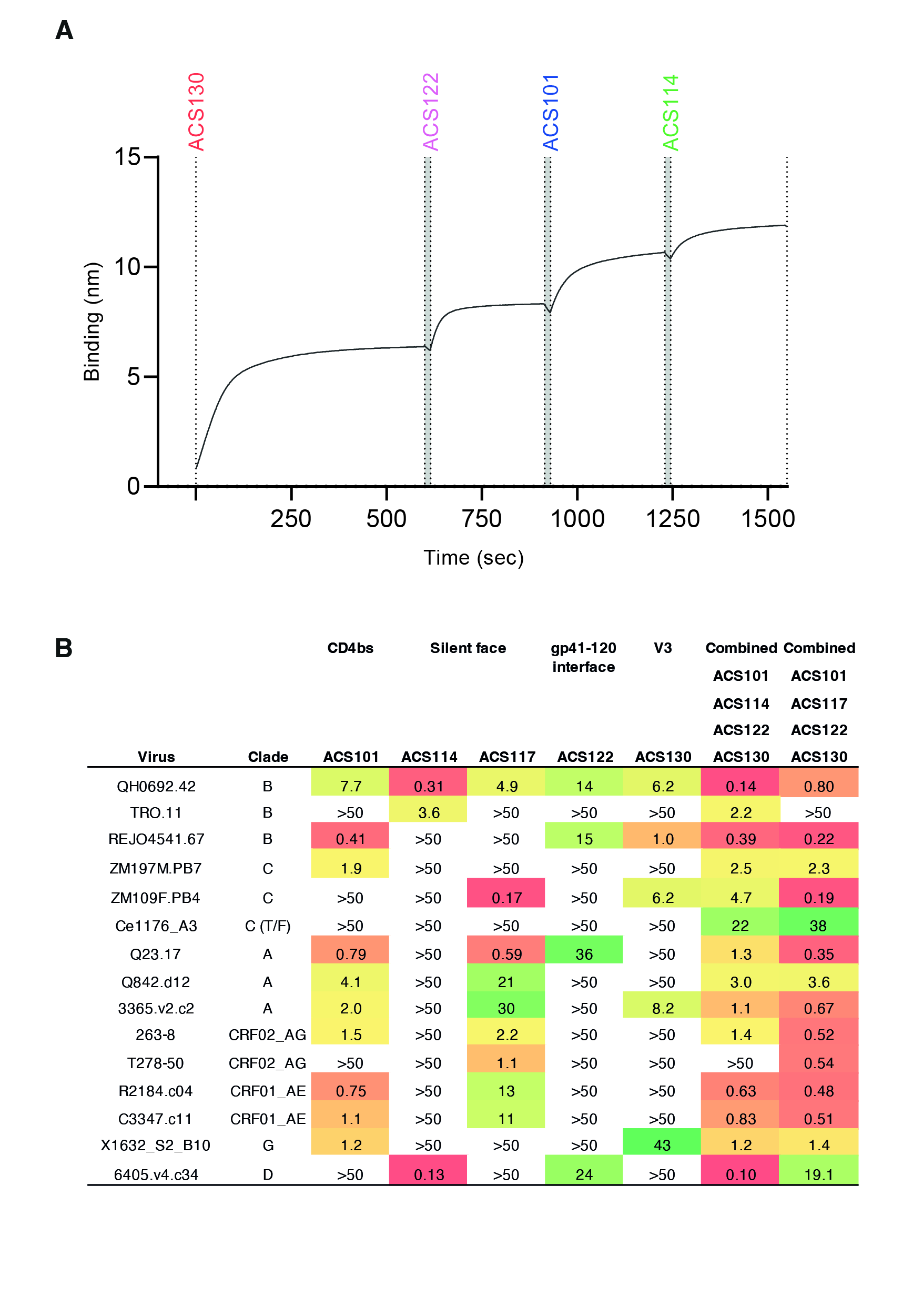

Supplement: S5 Fig — Combined binding and neutralization of antibody lineages (a) Simultaneous binding of ACS101, ACS114, ACS122 and ACS130 to AMC009 SOSIP trimer using bio-layer interferometry. (b) Neutralization of a multiclade 15-virus panel by ACS101, ACS114, ACS117, ACS122 and ACS130 individually or combined. The virus panel was based on positive hits from the large (n = 119) multiclade virus panel. Neutralization potency (antibody concentration (μg/ml) that inhibits 50% of viral infectivity (IC50) is given. (TIF) [file ppat.1010945.s005.tif]

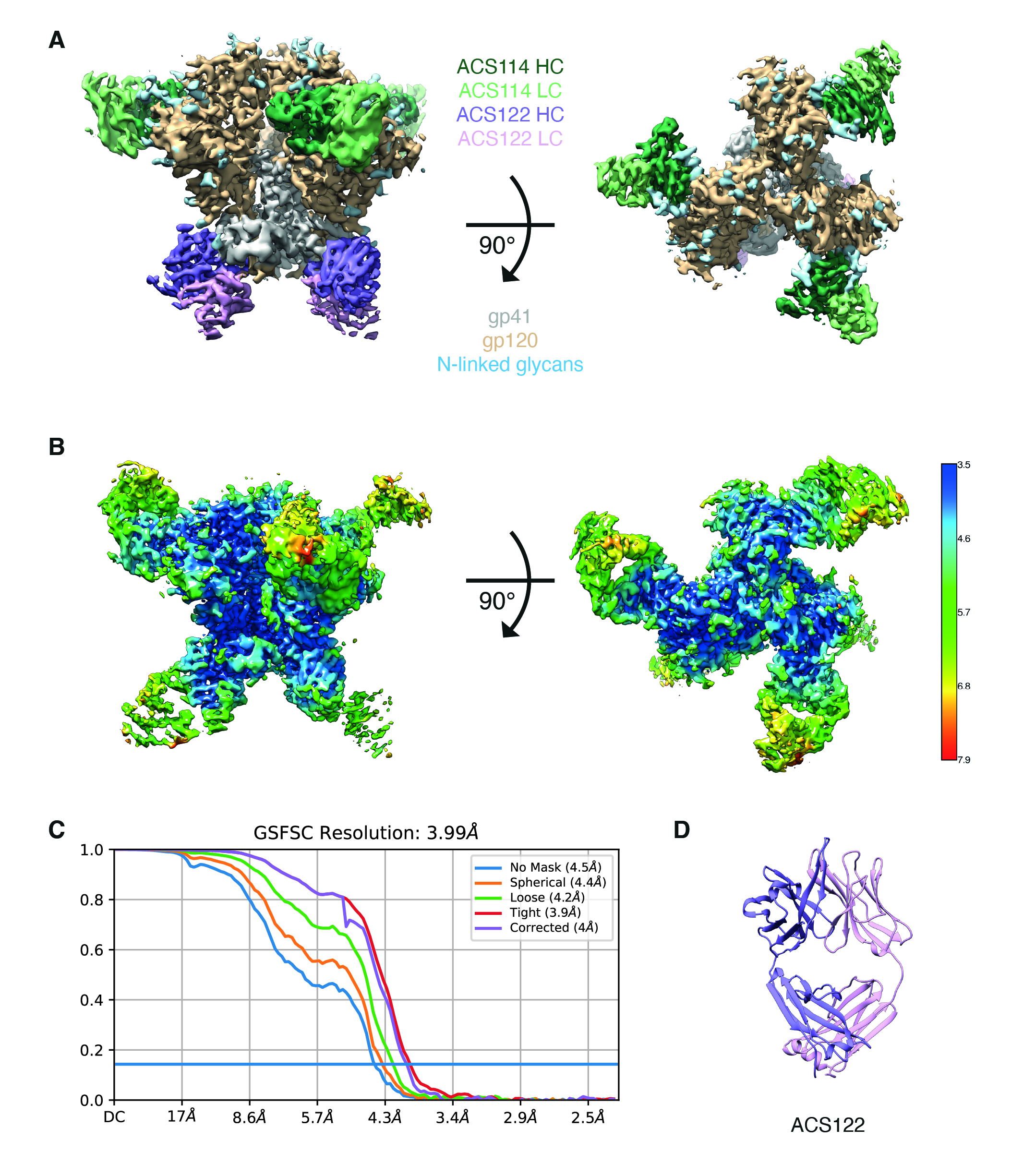

Supplement: S6 Fig — (a) Cryo-EM map of ACS122 and ACS114 in complex with the AMC009 SOSIP trimer. Only the Fab variable regions are shown here. (b) Cryo-EM map of ACS122 and ACS114 in complex with AMC009 SOSIP colored by local resolution (Å). (c) Gold-standard Fourier shell correlation (GSFSC) resolution estimate of ACS122 and ACS114 Fabs in complex with AMC009 SOSIP. (d) Crystal structure of unliganded ACS122 Fab (HC; purple and LC; light pink) at 1.84 Å resolution. (TIF) [file ppat.1010945.s006.tif]

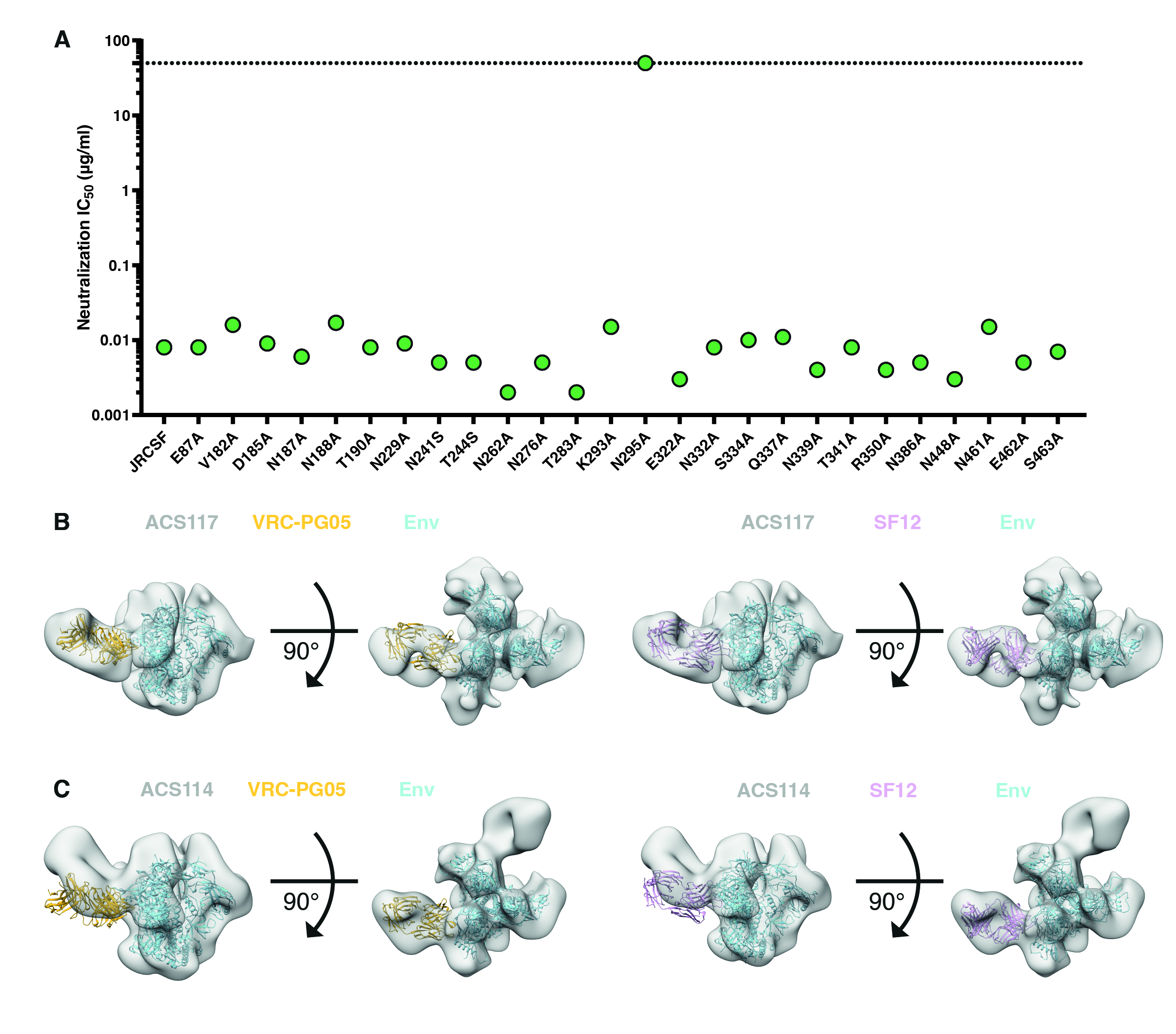

Supplement: S7 Fig — (a) Neutralization potency (antibody concentration (μg/ml) that inhibits 50% of viral infectivity (IC50) of ACS114 against a panel of JRCSF mutants. The different mutants are indicated along the horizontal axis. The mAbs were tested at a starting concentration of 50 μg/ml. (b) The NS-EM map of ACS117 with the structure of VRC-PG05 (PDB:6BF4) and SF12 (PDB:6OKQ) docked in. (c) The NS-EM map of ACS114 with the structure of VRC-PG05 (PDB:6BF4) and SF12 (PDB:6OKQ) docked in. (TIF) [file ppat.1010945.s007.tif]

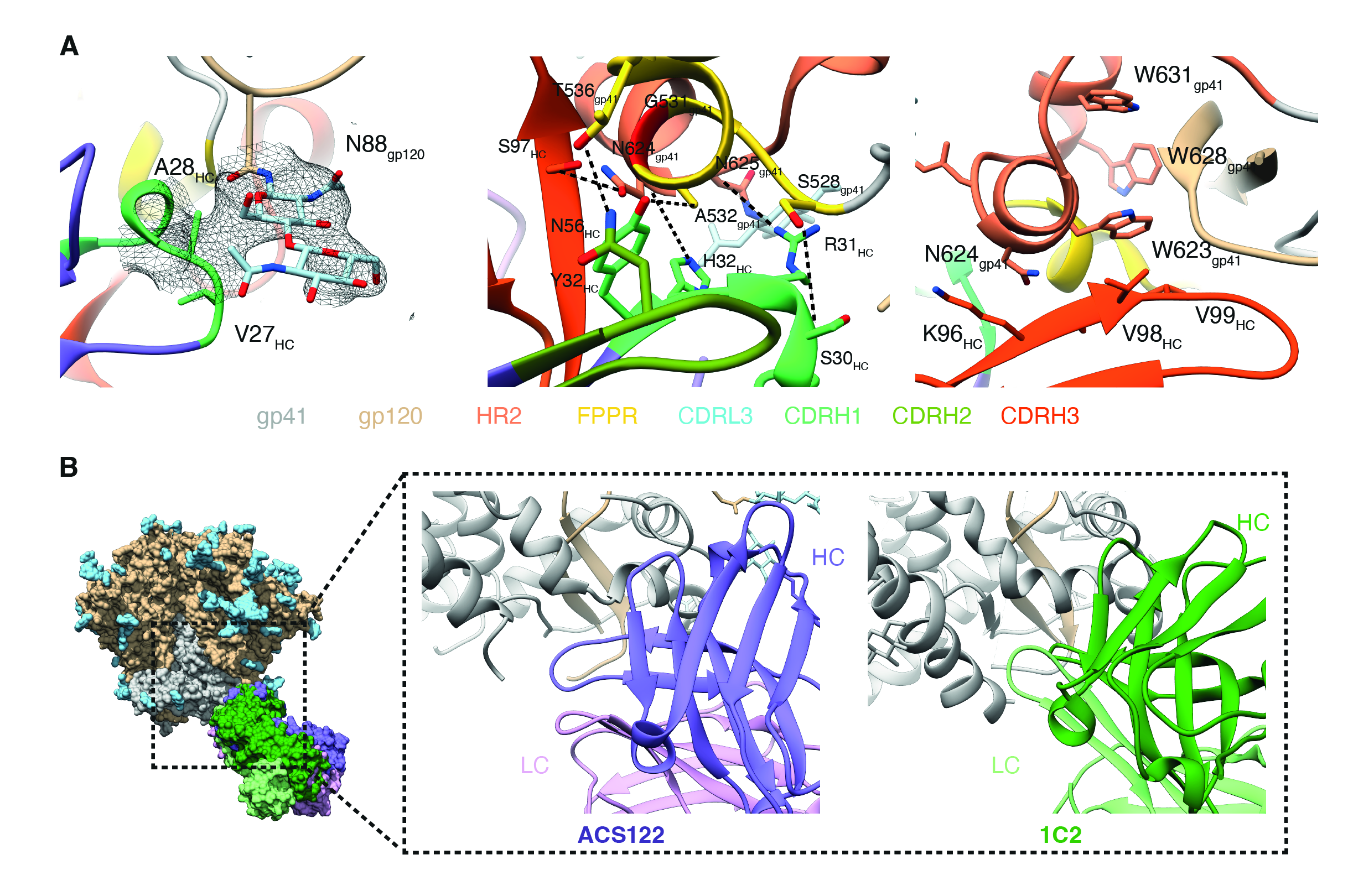

Supplement: S8 Fig — (a) Interactions of ACS122 with the N88gp120 glycan (left), FPPP (middle) and the tryptophan clasp and surroundings (right). The N88 glycan is shown as sticks and contoured by the cryo-EM map at 1σ. Amino acid interactions between ACS114 and gp120 are highlighted based on the density in the cryo-EM map and predicted hydrogen bonds are shown with a distance <3.2 Å. (b) Comparison of ACS122 to 1C2 (PDB:6PEH). Fabs and trimer are shown as a surface representation and the magnified view is depicted as ribbons. The structures were aligned relative to gp41. (TIF) [file ppat.1010945.s008.tif]

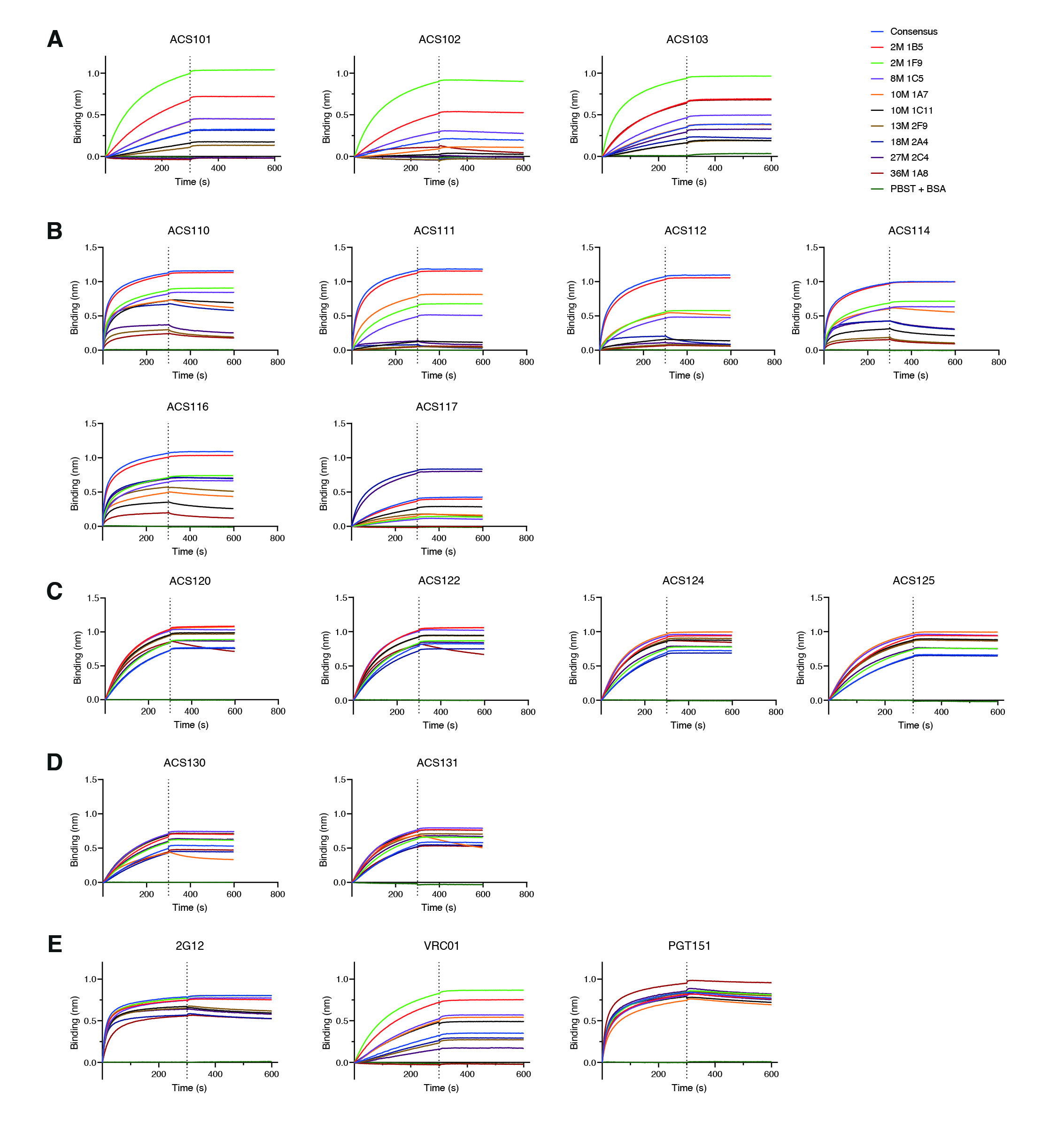

Supplement: S9 Fig — Binding curves are shown for mAbs targeting the (a) CD4-binding site, (b) silent face, (c) gp120-gp41 interface, (d) V3 region and (e) control bNAbs. Binding curves for different longitudinal Envs are colored as indicated on the right. (TIF) [file ppat.1010945.s009.tif]

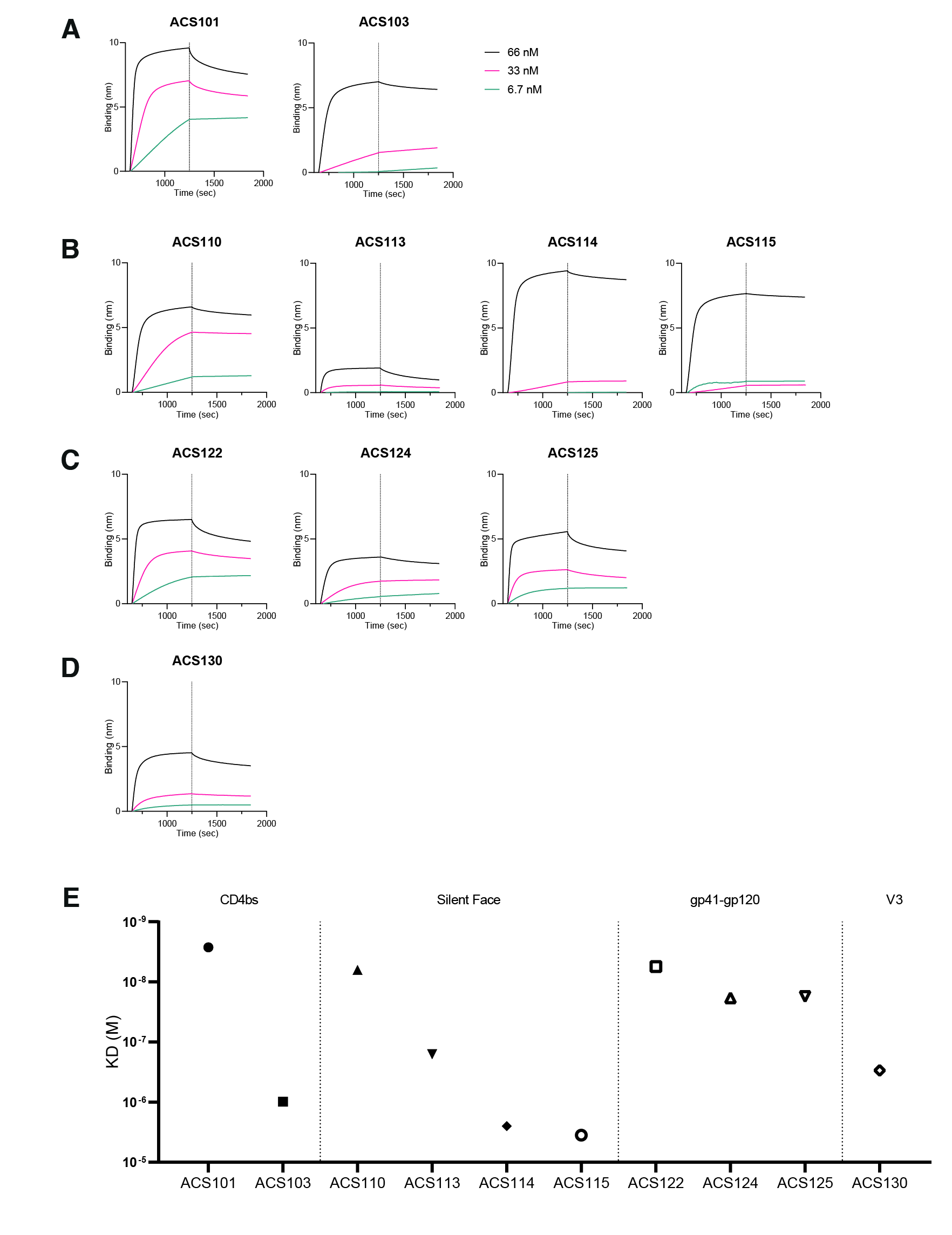

Supplement: S10 Fig — Binding curves are shown for various mAbs targeting the (a) CD4-binding site, (b) silent face, (c) gp120-gp41 interface, (d) V3 region. (e) Overview of the KD’s of the tested mAbs. Binding curves for mAbs with different concentrations are colored as indicated on the right. (TIF) [file ppat.1010945.s010.tif]

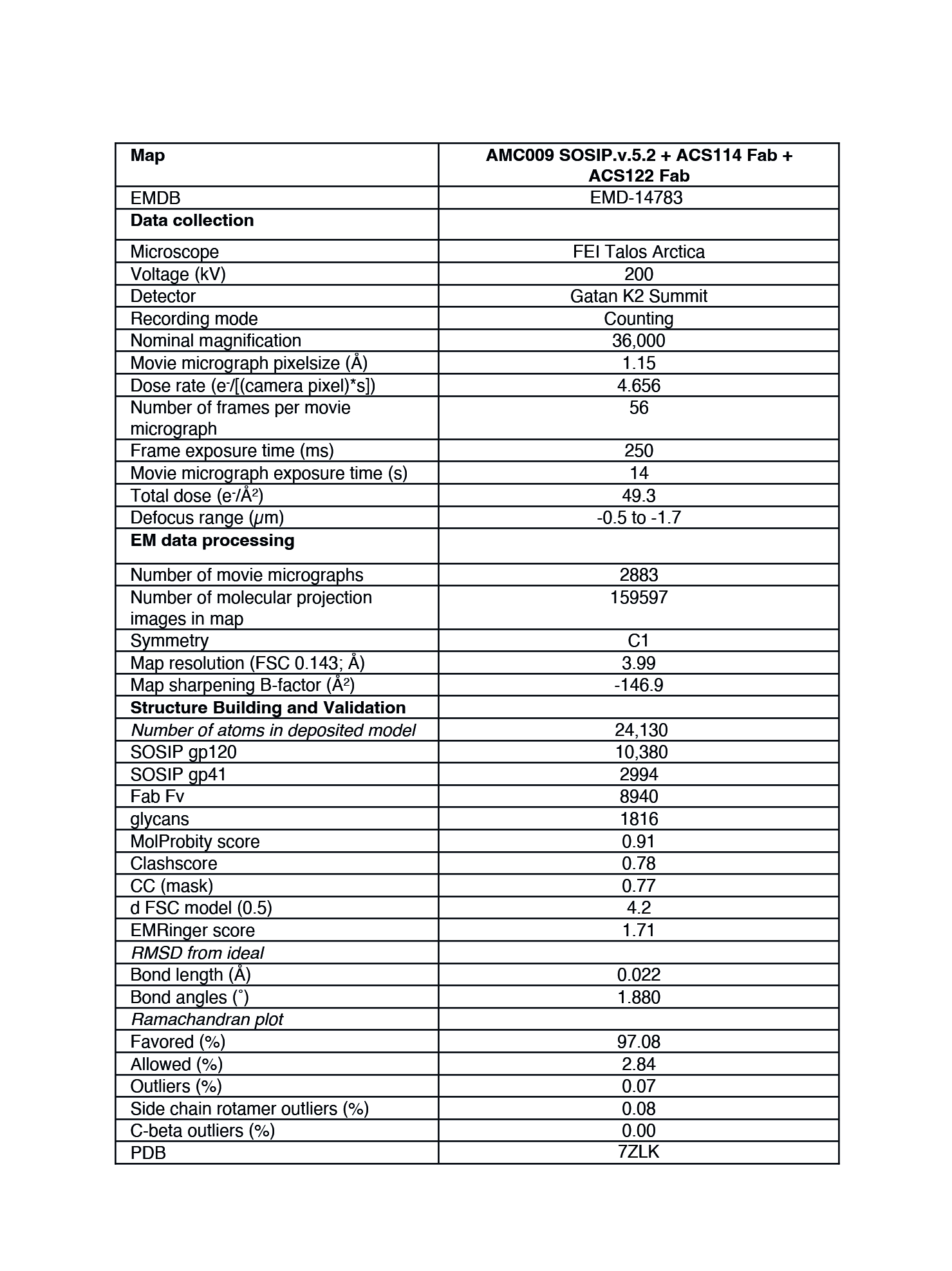

Supplement: S1 Table — (TIF) [file ppat.1010945.s011.tif]

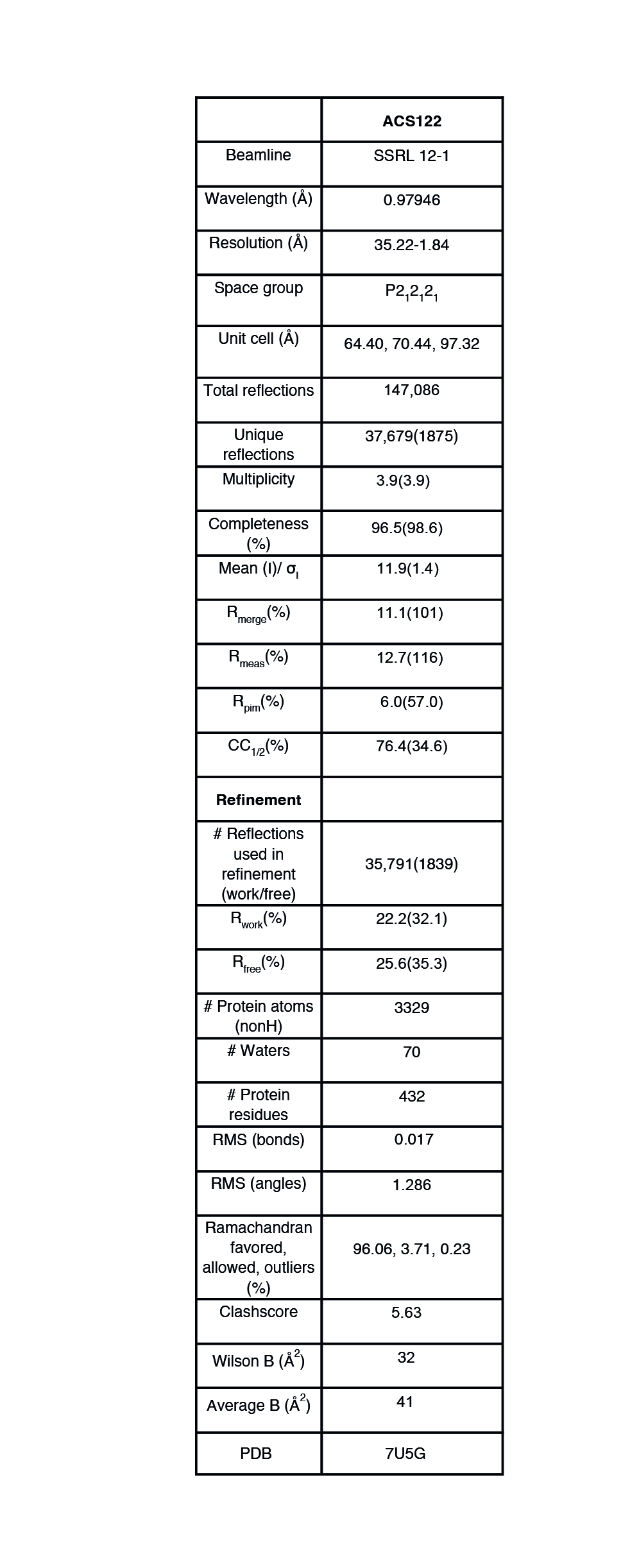

Supplement: S2 Table — Values in parentheses correspond to the highest resolution shells. (TIF) [file ppat.1010945.s012.tif]

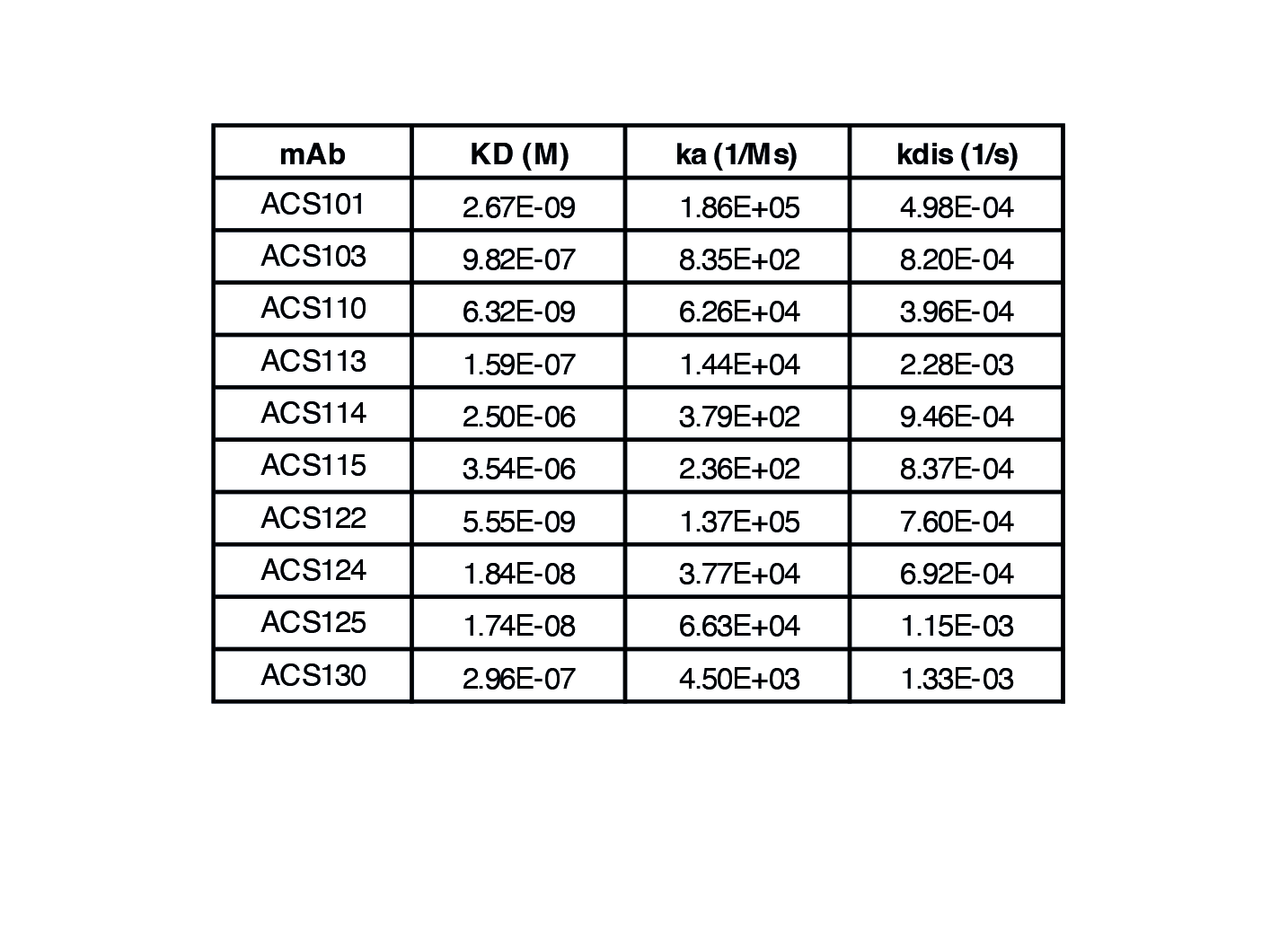

Supplement: S3 Table — (TIF) [file ppat.1010945.s013.tif]
