# Supplementary material for: Complementary antibody lineages achieve neutralization breadth in an HIV-1 infected elite neutralizer
Source: PLoS Pathog. 2022 Nov 17;18(11):e1010945. doi: 10.1371/journal.ppat.1010945 (PMC9714913; doi:10.1371/journal.ppat.1010945)
Supplement: Attachment — Submitted filename: van Schooten et al 2022 Response to reviewers.docx [file ppat.1010945.s014.docx]

We appreciate the time and effort the reviewers spent to review our manuscript. We are thankful for the constructive feedback, which will improve the quality of the manuscript. We tried to address each of the reviewers comments. The reviewers text is shown in italic black, our replies are in blue. Changes in the manuscript are shown in red.

***Part I - Summary*** *Please use this section to discuss strengths/weaknesses of study, novelty/significance, general execution and scholarship.
Reviewer #1: Schooten et al*

*To the editor

To the authors

The authors describe broadly neutralizing MAb isolation from an elite neutralizing HIV+ donor. They describe multiple bnAbs with various specificities that collectively do not fully recapitulate the breadth of the donor serum, and conclude that inducing combinations of moderate bnAbs could be a viable vaccine strategy.

The paper is another high capacity mAb recovery and detailed characterization study, which is by now a well-beaten path that this group and others have perfected in recent years. The authors then show that viral escape in the donor is driven by the two most potent lineages (CD4 binding site and silent face). Overall, the paper is well-written and succinct. A few minor comments and suggestions follow.*

We thank the reviewer for the accurate summary, kind words, and emphasizing the implications of this study to the field.
 *ACS130: the epitope of some mabs is called V1V2V3, but further studies suggest that it is V3 directed, so this may be a “straw man” for review? It seems possibly slightly misleading to say it is V1V2V3 in the earlier parts of the paper including the abstract as this gives the impression of a new complex V1V2V3 epitope or possibly V1V2 apex epitope.*

This a valid argument and we now have changed the V1V2V3 epitope/region to a V3-directed epitope, or V3 region, in the text and figures of the manuscript.

*Line 110, 152, 220. To say “we previously described…” then to cite a paper that is not published seems contradictory unless this other paper is published while this one is in review.*
The manuscript that we referred to was in press at the time that we submitted the current manuscript to PloS Pathogen. The related manuscript has now been published in Nature Communication. We have removed all (Jelle van Schooten et al. 2022, submitted) references and now correctly refer to the published manuscript:

26. van Schooten, J. et al. Identification of IOMA-class neutralizing antibodies targeting the CD4-binding site on the HIV-1 envelope glycoprotein. Nat. Commun. 2022 131 **13**, 1–15 (2022).

*Line: 139-140. I am not sure if it is OK to say ‘data not shown’ for this journal?*
We have removed ‘data not shown’ from the text and described the results. *The interface mAbs show weak neutralizing titers >10ug/ml, compared to some of the known ones. These ones also exhibit the least breadth, although the breadth and potency of the other Mabs is not particularly high, hence the suggestion that they be used together. Do the authors know what might make these MAbs less potent than some of the published bnAbs? For example, do multiple lineages mean there is less successful SHM in each as compared to when fewer lineages are developing? Or are these mabs simply less potent versions of other mabs in the donor that have not been mined out of the donor yet? Is it true that broadly neutralizing donors always harbor lineages with a range of breadth and potency, including weaker ones, as compared to a few potent and broad mabs, as prior studies imply?*
We regret that this was not clear from the text, and now give various reasons that could explain why ACS122 is less potent and broad compared to the prototype gp41/gp120 interface bNAb 35O22. Firstly, ACS122 CDRH3 makes extensive contact with N624 which is only found on 31.54% of HIV-1 viruses (lines 251-254). Secondly, ACS122 binds to Env with a different angle of approach then the broad gp41/gp120 interface bNAb 35O22 which may explain the difference in breadth/potency (lines 268-269). Thirdly, ACS122 contains significantly lower levels of SHM compared to 35O22 and lacks the 8 amino acid insertion in the framework 3 that characterizes 35O22 (lines 270-277).

Broadly neutralizing antibodies develop after years of HIV infection, and therefore undergo extensive somatic hypermutation to achieve their breadth and potency. In this process various clonal family members are generated that contain fewer SHM which are less potent and broad. Eventually, family members will acquire accumulated SHM levels after years of infection that will lead to their potency and neutralization breadth. The neutralization breadth members of the lineages that we have isolated do not fully recapitulate the serum’s breadth. Therefore, it is likely that we have missed more potent members of these lineages, or missed other lineages that were present in the serum. This is stated in lines 362-364.

*Line 253. Why would a 73-561 disulfide mitigate mab induced protomer dissociation? A lateral disulfide might prevent it, though. Does the interface mab cause dissociation of the real virus trimers?*
We agree with the reviewer that a lateral disulfide would prevent protomer dissociation better than the A73C-A561C disulfide bond. We have removed the sentence ‘’despite the use of the SOSIP.v5.2 design that includes an additional disulfide bond (A73C-A561C) to improve stability’’ from the text. We now have added ‘’Dissociation of the AMC009 SOSIP trimer did not occur spontaneously when the trimer was left overnight at room temperature in the absence of ACS122. This suggest that the Fab was responsible for the observed trimer dissociation’’ to clarify that the Fab is responsible for trimer dissociation (lines 256-259).

We also agree that experiments showing that ACS122 causes dissociation of Env trimers on viruses would be more informative to pin-point the exact mechanism of neutralization even better. These experiments would have required substantial amount of additional work and we were therefore unable to perform these experiments without substantial delay of this manuscript. We think that these data are not essential to the message in this manuscript. We believe that the NS-EM data (protomer dissociation) and cryo-EM structure (showing that ACS122disrupts the tryptophan clasp) suggest that this is the mechanism of neutralization of these mAbs. In addition, in the text we use ‘’may destabilize’’ (lines 278) and ‘’might promote trimer dissociation’’ (lines 385) to illustrate that we cannot say with a hundred percent certainty that this also happens on the virus.

*Line 309. The text talks about mutations in the epitope of the interface mAb, but the mAb binds equally to them. Nevertheless, the authors say that the mutations indicate when the mAb was generated at 2-8 months. However, since the mutations, although close to the epitope don’t impact binding per se, I don’t follow the reasoning, as no “escape” seems to be.*

We agree with the reviewer that the interface mAbs bind equally to the longitudinal SOSIPs and that it is therefore hard to conclude that the virus escapes from the gp120-gp41 mAbs. We have now stated this in lines 332-334. We have changed the text in the manuscript and now state that although the mAbs bind equally to longitudinal SOSIPS, changes in the amino acids close to their epitope may indicate escape of other family members of this lineage (lines 335-336).

*Reviewer #2: The manuscript by van Schooten and colleagues identified and characterized three complementary bNAb lineages from an elite neutralizer, which targeted non-overlapping Env epitopes, including the silent face, gp120-gp41 interface, and V1/V3 region. The authors performed comprehensive biochemical and structural analyses of longitudinal samples to provide the basis of antibody neutralization and env evolution. While no single antibody lineage recapitulated the complete plasma neutralizing activity, the polyclonal mixture of select antibodies from these three lineages (plus a CD4bs lineage identified from the same donor and described in a related manuscript) was able to yield potent and broad neutralizing activity. Thus, the authors postulate that vaccine strategies capable of eliciting multiple antibody lineages that target complementary Env epitopes with moderate breadth and potency is an achievable goal. While the studies in this manuscript are well carried out and written, there are some points the authors should address in a revised manuscript prior to publication.*

We thank the reviewer for recognizing the implications of this study for the field and for providing constructive feedback to improve the manuscript.

***Part II – Major Issues: Key Experiments Required for Acceptance*** *Please use this section to detail the key new experiments or modifications of existing experiments that should be absolutely required to validate study conclusions.

Generally, there should be no more than 3 such required experiments or major modifications for a "Major Revision" recommendation. If more than 3 experiments are necessary to validate the study conclusions, then you are encouraged to recommend "Reject".*

*Reviewer #1: (No Response)*

*Reviewer #2: The authors performed neutralization studies of antibodies, but missed an opportunity to provide critical data on the effect of affinity to Env epitopes for antibodies within the same lineage isolated at different timepoints. For example how does ACS110 affinity compare to ACS114?*

We agree that affinity measurements of the antibodies provide a better overview of the evolution of antibodies within the lineages that are isolated at different timepoints. However, since the AMC009 SOSIP and longitudinal SOSIP trimers did not contain any tags, we were restricted to first couple the IgG’s to the bio-layer interferometry sensors instead of the SOSIP trimers. Therefore, we were unable to determine affinities using the described bio-layer interferometry set-up because of potential avidity affects. Therefore, to assess the affinities of these IgGs against all longitudinal SOSIPs, it would require a significant amount of additional work by cloning all of the SOSIP trimers into new vectors that contain tags. Therefore, we have chosen to only re-clone the AMC009 SOSIP consensus in to a his-tagged construct. We performed affinity measurement using bio-layer interferometry with AMC009 SOSIP consensus and various mAbs within the same lineages isolated at different timepoints (Fig S10 and Table S3). The results show that members isolated at later timepoints bind with higher affinities to AMC009 SOSIP. This indicates that the mAbs isolated at later timepoints likely originated from B cell descendants that developed later within the lineages, which is now explained in the manuscript in lines 341-343.

*Additionally, at timepoints 2 and 8 mo seroconversion the authors suggest in lines 299-300 and 314 that this is when the silent face and gp41/gp120 lineages developed. Did the authors observe intermediate antibodies with less somatic mutation at these timepoints for those lineages? If so, how do the affinities of these antibodies compare to the mature neutralizing antibodies? What might this say about the level of SHM necessary to elicit neutralizing antibodies?*

Unfortunately, we were unable to isolate more family members also from timepoints 2- and 8-months post-SC. The earliest antibodies were isolated at 11 months post-SC, and we can therefore not say anything about SHM in these lineages before 11 months post-SC. Based on the mutations in the epitopes of these lineage around months 2 and 8 post-SC, we speculate that this is when these lineages developed.

*Additionally, the authors show that when combined, there is no synergy between the different antibody classes. However, outside of the ACS114-ACS122-Env structure, the authors do not show if the antibodies can target Env simultaneously. The authors should provide competition BLI or ELISA binding data, which may point at the inability of antibodies to synergize.*

We performed an additional biolayer interferometry experiment and are now able to show that antibodies from every lineage can bind AMC009 SOSIP simultaneously (Fig. S5A) (lines 203-204). We immobilized AMC009 SOSIP on the sensor, and subsequently loaded ACS101, ACS114, ACS122 and ACS130 to the sensor (one antibody from every lineage). All four antibodies could bind to the same trimer simultaneously. *Given the finding that ACS122 binds Env in a similar way to 1C2 and also disrupts the tryptophan clasp, it’s surprising that the ACS122 doesn’t show broad neutralizing activity. Antibody 1C2 was capable of moderately neutralizing 87% of isolates in a 208-virus panel. A more thorough comparison of ACS122 and 1C2 would be appreciated, particularly, what are the major differences that give rise to broad neutralization for 1C2, but limit ACS122?*

We have added a possible explanation on why ACS122 is less broad compared to 1C2, and also 35O22, also see our response to a similar comment from reviewer 1 (lines 268-277).

***Part III – Minor Issues: Editorial and Data Presentation Modifications*** *Please use this section for editorial suggestions as well as relatively minor modifications of existing data that would enhance clarity.*

*Reviewer #1: (No Response)*

*Reviewer #2: For silent face antibodies, removal of glycans at position 295 or 448 disrupted binding. However, env sequences in the C4 region included addition of a glycan at position 444, which would appear to clash with ACS114 binding. However, BLI data against Env sequence 27m.2C4 shows binding in the presence of the N442-glycan. Could the authors speculate how ACS114, in particular, can accommodate this glycan?*
This is an interesting point that we have now incorporated in the manuscript (lines 309-314). We speculate that ACS114 can still bind to the 27m.2C4 because the N444 PNGS may be under-occupied. A previous study (Derking, R. et al. Enhancing glycan occupancy of soluble HIV-1 envelope trimers to mimic the native viral spike. Cell Rep. 35, (2021)) has shown that PNGS in close proximity affect their occupancy on SOSIP trimers. As a result, the N444 PNGS may be underoccupied on the 27m.2C4 trimer and therefore ACS114 can still bind. *Line 101: “..published results of the AMP trials implies..” should be “imply.”*

We have changed ‘’implies’’ to ‘’imply’’ in the text (line 102).

*Line 162: “In addition, we isolated an eight member..” should be “eighth member.”*

We have changed ‘’eight’’ to ‘’eighth’’ in the text (line 161).

*Line 218: “.. HIV-1 vaccine design how to induce similar..”, should remove “how”*

We have removed ‘’how’’ from the text (line 219)

*Line 250: “Furthermore, ACS112 CDRH1..”, should this be ACS122?*

We agree with the reviewer that this was a typo and we have corrected ACS112 to ACS122 (line 249).
